# Schistosomiasis Presenting as a Case of Acute Appendicitis with Chronic Mesenteric Thrombosis

**DOI:** 10.1155/2016/5863219

**Published:** 2016-05-10

**Authors:** Mohammed H. Mosli, Wilson W. Chan, Izabella Morava-Protzner, Susan M. Kuhn

**Affiliations:** ^1^Department of Community Health Sciences, Faculty of Medicine, University of Calgary, Teaching, Research & Wellness (TRW) Building, Room 3D03-4 (3rd Floor), 3820 Hospital Drive NW, Calgary, AB, Canada T2N 4Z6; ^2^Department of General Medicine and Clinics, King Abdulaziz University Hospital, Jeddah, Saudi Arabia; ^3^Calgary Laboratory Services, Calgary, AB, Canada; ^4^Department of Pathology & Laboratory Medicine, University of Calgary, Calgary, AB, Canada; ^5^Department of Pediatrics, University of Calgary, Calgary, AB, Canada; ^6^Alberta Children's Hospital, Calgary, AB, Canada

## Abstract

The manifestations of schistosomiasis typically result from the host inflammatory response to parasitic eggs that are deposited in the mucosa of either the gastrointestinal tract or bladder. We present here a case of a 50-year-old gentleman with a rare gastrointestinal presentation of both schistosomal appendicitis and mesenteric thrombosis.

## 1. Introduction

Schistosomiasis is a parasitic disease that afflicts approximately 250 million people worldwide [1]. Human infection is caused by* S. mansoni*,* S. haematobium*,* S. japonicum,* and* S. mekongi*, all of which have similar life cycles. Transmission to humans occurs when cercariae, the infective stage of the parasite, penetrate the host's skin and develop into schistosomula. These then migrate to the portal vein where they mature into adult forms, further proceeding to either the pelvic (*S. haematobium*) or mesenteric venules (*S. mansoni* and* S. japonicum*) where they mate and produce eggs [1]. Without treatment, schistosomes survive in the human body for an average of four and a half years but may persist up to forty years [2].

Chronic infection typically results in a progressive illness due to prolonged tissue injury and fibrosis caused by host inflammatory responses to eggs deposited in affected organs, usually intestine and liver for* S. mansoni* and* S. japonicum* and bladder, kidney, and ureters for* S. haematobium*. Two unusual presentations of gastrointestinal schistosomiasis are appendicitis and chronic recurrent epigastric pain due to mesenteric thrombosis. We present here a case where both of these rare presentations occurred concurrently.

## 2. Case Presentation

Our patient is a 50-year-old, previously healthy male who recently immigrated to Canada from the Philippines. He presented to the emergency department with acute right iliac fossa pain that he had been experiencing for several days. The pain was not associated with nausea or vomiting, and he denied any significant headache, cough, diarrhea, hematochezia, dysuria, urinary frequency, myalgia, arthralgia, or rash. There was a history of a similar episode six months prior to the current presentation; however, it resolved after one day and he did not seek medical care.

On examination, he was found to have tenderness localized to the right lower quadrant (RLQ) of the abdomen which was otherwise unremarkable. A complete blood count showed normal haemoglobin (142 g/L) and leukocyte count (71 × 10^9^/L) without eosinophils; platelets were low at 44 × 10^9^/L. C-reactive protein was significantly elevated at 68.3 mg/L. Creatinine, alanine transaminase, lactate dehydrogenase, and lipase were normal, but his total bilirubin (31 *μ*mol/L), alkaline phosphatase (168 U/L), and *γ*-glutamyl transferase (139 U/L) were elevated. Cultures of blood and urine showed no growth.

The initial clinical impression of his presentation was acute appendicitis. An ultrasound showed nonspecific RLQ intraperitoneal inflammatory changes in the region of the terminal ileum. CT scan subsequently demonstrated a thick-walled and hyperemic appendix, as well as a clot in the superior mesenteric vein (SMV). A final diagnosis of SMV thrombosis concurrent with the acute appendicitis was made. As such, the patient was managed conservatively with anticoagulation and intravenous antibiotics for six days. He was then discharged on oral warfarin with a plan to follow up with the anticoagulation clinic for further workup.

A month later, the patient presented again with recurrence of the RLQ pain for 36 hours. Repeat imaging by CT showed an increase in inflammatory changes around the appendix, including thickening of its wall along the cecum and adjacent loop of small bowel. No clot was evident in the SMV. The patient underwent an urgent appendectomy, after his anticoagulation was reversed, and IV ceftriaxone and metronidazole were administered. Postoperatively, his recovery was uneventful and he was discharged home on oral antibiotics.

**Figure 1:**
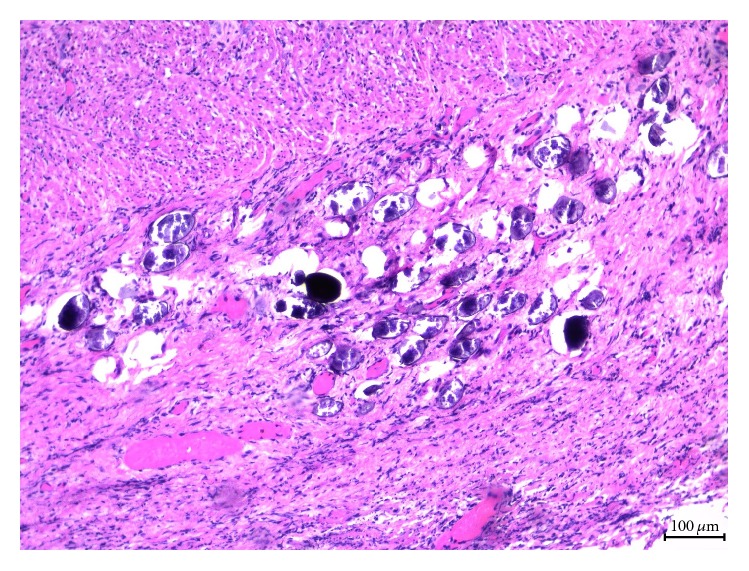
Ova of* S. japonicum* seen throughout the appendiceal lumen, wall, and periappendiceal adipose tissue accompanied by acute inflammation.

Histopathological examination of the excised appendix revealed acute inflammatory changes, marked with numerous ova identified within the appendiceal lumen, wall, and periappendiceal adipose tissue. Further review of the specimen by microbiology confirmed that these were* Schistosoma* ova which were described as approximately 60 *μ*m in length, ovoid to spherical in shape, and with no spines visualized on any of the eggs (Figure 1). The ova were numerous, and many showed signs of degeneration and calcification (Figure 1). These features were suggestive of* S. japonicum*. Stool was examined for ova and parasites but was negative.

The patient was referred to the tropical disease clinic for further assessment and treatment. Further questioning revealed that the patient was born and had lived most of his life on the island of Samar, in the city of Borongan. He made his living both as a fisherman and a rice farmer and therefore had extensive freshwater contact. Our patient also confirmed that numerous family members and coworkers in Borongan were diagnosed with and treated for schistosomiasis. He was prescribed 60 mg/kg/d of praziquantel divided into 3 doses which he tolerated well.

## 3. Discussion

As the third most prevalent parasitic disease globally after malaria and soil-transmitted helminths, schistosomiasis is a disease that is well recognized in developed countries [3]. However, due to human schistosomal species being nonindigenous in North America, cases are relatively rare. Nevertheless, with the steady growth in immigration from developing to industrialized nations, more cases of chronic schistosomiasis have been reported [4].

Prevalent in East Asian countries,* S. japonicum* is a recognized public health challenge in the Philippines, China, and Indonesia [1]. Santos estimated that over 800,000 individuals in the Philippines have active* S. japonicum* infection. The majority of those infected were in the islands of Leyte, Samar, and Mindanao [5].

Chronic schistosomiasis causes multisystem complications that are well described in the literature [6]. The incidence of schistosomal appendicitis in Japan has been reported to be 0.34% compared to 0.29% in the United States and 4.2% of resected appendices in Nigeria. Other gastrointestinal complications including portal hypertension, esophageal varices, liver cirrhosis, and rectal bleeding have also been described [6]. However, reports describing superior mesenteric vein thrombosis as a recognized complication of* S. japonicum* remain scarce [7].

Generally, prothrombotic states such as surgery, inflammatory bowel disease, and malignancy can be suspected in any thrombotic phenomenon. However, risk factors for thrombotic events specifically involving the mesenteric vein have been commonly seen secondary to local factors, such as adjacent malignancy, pancreatitis, or infection [8]. When the thrombus is large enough to block the SMV, ischemia starts developing and approximately 75% of patients present with findings suggestive of acute SMV thrombosis such abdominal tenderness, abdominal distention, and ascites. In contrast, chronic SMV thrombosis rarely presents so prominently and is typically an incidental finding on abdominal imaging [8].

The ambiguity in presentation of SMV thrombosis is significantly different from that of acute appendicitis, which is a highly probable diagnosis in any patient presenting with acute RLQ pain. Although our patient was initially found to have symptoms suggestive of appendicitis, further CT imaging revealed a thrombus which prevented a definitive surgical treatment of acute appendicitis. As such, the precautionary approach of conservatively treating him with anticoagulation and antibiotics temporarily ameliorated his appendiceal symptoms without curing the cause of appendicitis.

## 4. Conclusion

We present the first case of schistosomiasis-associated appendicitis with superior mesenteric vein thrombosis. Schistosomiasis is a major worldwide concern due to its high prevalence in many developing countries and has a spectrum of clinical presentations. However, with increasing global migration and cross-border travel, schistosomiasis should be considered as a cause of acute appendicitis, especially among immigrant patients coming from endemic areas or patients with a history of travel to regions where schistosomiasis is common.
